# Supplementary figures and images for: Brain-related genes are specifically enriched with long phase 1 introns
Source: PLoS One. 2020 May 29;15(5):e0233978. doi: 10.1371/journal.pone.0233978 (PMC7259759; doi:10.1371/journal.pone.0233978)

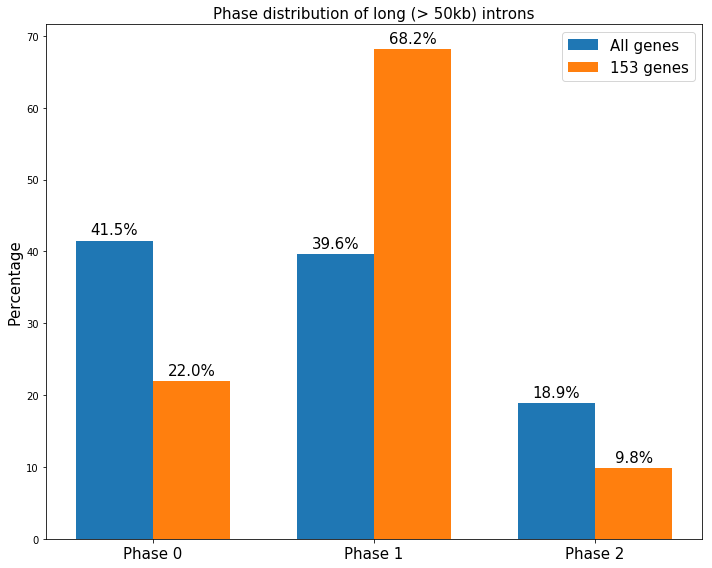

Supplement: S1 Fig — (PNG) [file pone.0233978.s001.png]

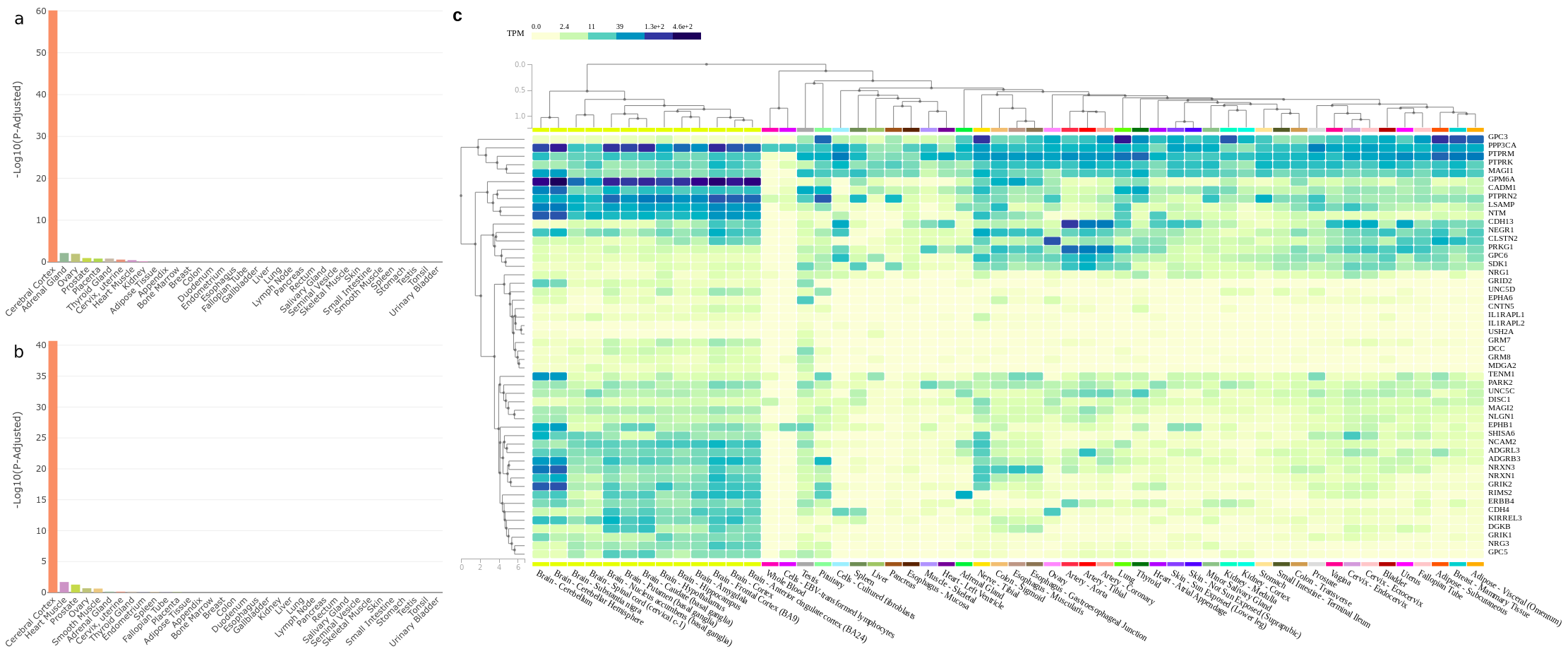

Supplement: S2 Fig — (a) All 507 genes containing ph1>50 introns. (b) 153 genes selected based on GO enrichment. Graphs have been generated with TissueEnrich [1]. (c) The top 50 genes (out of the 153 genes) with the largest total phase 1 introns size. The graph is generated with GTEx Multi Gene Query tool [2]. (PNG) [file pone.0233978.s002.png]

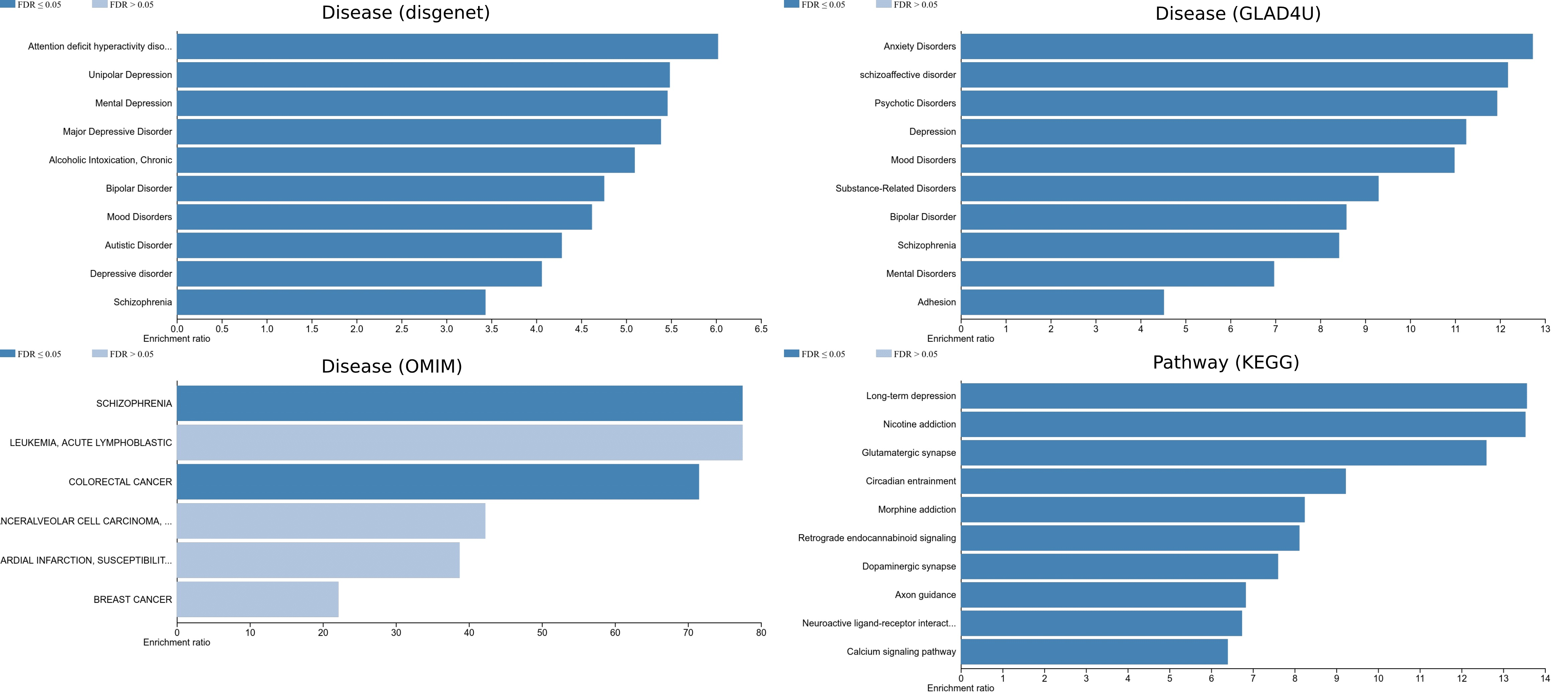

Supplement: S3 Fig — Graphs have been generated with WebGestalt [3]. (PNG) [file pone.0233978.s003.png]
